# Simulating Land-Use Changes and Predicting Maize Potential Yields in Northeast China for 2050

**DOI:** 10.3390/ijerph18030938

**Published:** 2021-01-22

**Authors:** Luoman Pu, Jiuchun Yang, Lingxue Yu, Changsheng Xiong, Fengqin Yan, Yubo Zhang, Shuwen Zhang

**Affiliations:** 1 School of Politics and Public Administration, Hainan University, Haikou 570228, China; 994424@hainanu.edu.cn (L.P.); xiongcs@hainanu.edu.cn (C.X.); 2 Northeast Institute of Geography and Agroecology, Chinese Academy of Sciences, Changchun 130102, China; yulingxue@iga.ac.cn (L.Y.); ybzhang19@mails.jlu.edu.cn (Y.Z.); 3 State Key Laboratory of Resources and Environmental Information System, Institute of Geographic Sciences and Natural Resources Research, Chinese Academy of Sciences, Beijing 100101, China; yanfq@lreis.ac.cn; 4 College of Earth Sciences, Jilin University, Changchun 130021, China

**Keywords:** CA-Markov model, GAEZ model, land-use changes simulation, maize potential yields, food security, Northeast China

## Abstract

Crop potential yields in cropland are the essential reflection of the utilization of cropland resources. The changes of the quantity, quality, and spatial distribution of cropland will directly affect the crop potential yields, so it is very crucial to simulate future cropland distribution and predict crop potential yields to ensure the future food security. In the present study, the Cellular Automata (CA)-Markov model was employed to simulate land-use changes in Northeast China during 2015–2050. Then, the Global Agro-ecological Zones (GAEZ) model was used to predict maize potential yields in Northeast China in 2050, and the spatio-temporal changes of maize potential yields during 2015–2050 were explored. The results were the following. (1) The woodland and grassland decreased by 5.13 million ha and 1.74 million ha respectively in Northeast China from 2015 to 2050, which were mainly converted into unused land. Most of the dryland was converted to paddy field and built-up land. (2) In 2050, the total maize potential production and average potential yield in Northeast China were 218.09 million tonnes and 6880.59 kg/ha. Thirteen prefecture-level cities had maize potential production of more than 7 million tonnes, and 11 cities had maize potential yields of more than 8000 kg/ha. (3) During 2015–2050, the total maize potential production and average yield decreased by around 23 million tonnes and 700 kg/ha in Northeast China, respectively. (4) The maize potential production increased in 15 cities located in the plain areas over the 35 years. The potential yields increased in only nine cities, which were mainly located in the Sanjiang Plain and the southeastern regions. The results highlight the importance of coping with the future land-use changes actively, maintaining the balance of farmland occupation and compensation, improving the cropland quality, and ensuring food security in Northeast China.

## 1. Introduction

Land resources are the essential material basis for human survival and development, and the most precious natural resources. Since the beginning of the 21st century, with the rapid development of China’s industrialization and urbanization, as well as the increasing population, human utilization of land resources has been intensified, and the process of land-use changes has been accelerated [1]. The acceleration of urbanization and industrialization has led to a series of social, ecological, and environmental problems, as well as the rapid change of cropland resources [2]. On the one hand, the amount of cropland resources is seriously threatened. According to the statistical data, China’s urbanization and industrialization occupy 0.63 million ha of high-quality cropland resources every year [3]. On the other hand, the frequent changes of cropland and other land-use types makes it difficult to guarantee the cropland quality. Under the dual threat of the decrease in the cropland quantity and quality, the grain production capacity has been greatly affected, aggravating the contradiction between the cropland protection and food security and the social development [4].

At present, China is experiencing rising demands for grain production. Ensuring national food security has always been the foundation of China’s economic development, national independence, and long-term stability. Cropland is the natural carrier of grain production. Although China’s grain yields have been continuously improving in recent years, increasing by more than 600 kg/ha from 2010 to 2018, it is at the cost of high input, high cost, and sacrifice of ecological environment to some extent [1]. If human beings continue to occupy high-quality cropland and neglect the improvement of cropland quality, it will be difficult to guarantee China’s food security in the future. Therefore, to satisfy the growing population and food demand, it is greatly significant to estimate the future land-use changes and further predict the future crop potential yields.

In recent decades, the simulation of future land-use changes has become a major research topic. Land-use simulation models have been powerful tools to analyze the process of land use/land cover (LULC) change and to simulate future land use [5]. Many scholars have explored various models, which can be divided into three types: quantitative prediction models, spatial prediction models, and quantitative spatial coupling prediction models. Quantitative prediction models and spatial prediction models were popular before 2010. Quantitative prediction models were mainly used to predict the area of future land-use types. The principles were to use a mathematical method to calculate the results by putting the data into the formula, such as a Markov model [6,7,8], Logistic model [9,10,11], etc. Markov is a method to predict future land-use changes based on the quantities of land-use types and land-use transition matrix in two historical years. Aaviksoo (1995) used the Markov model to simulate vegetation dynamics and land use in marsh landscapes [6]. Wu et al. (2006) examined the time stability and time independence of a Markov chain of land-use change based on the land-use change data monitored in Beijing, and with a Pearson chi-squared goodness-of-fit test [7]. The Logistic regression model identifies the significant correlation between the driving factors and land-use changes through quantitative analysis, so as to predict the probability of the occurrence of a certain land-use type under current circumstances. Gobin et al. (2002) used the Logistic regression model to accurately predict the agricultural land in Southeastern Nigeria [9]. Spatial prediction models mainly explore the spatial variation rules of land-use types, such as the Cellular Automata (CA) model [12,13] and the Conversion of Land Use and its Effects at Small regional extent (CLUE-S) model [14,15]. The CA model is a kind of model that simulates the more complex spatial structure in the future through simple local transition rules. The CLUE-S model was developed by scholars and represented by Verburg to improve the CLUE model, which simulated land-use changes in large-scale regions [14]. It is a dynamic and multi-scale simulation model of land-use spatial distribution. The principle of CLUE-S model is to input data of various land-use types and driving factors into the model to simulate the spatio-temporal dynamics of land use. Since land-use changes are the spatio-temporal dynamic process, it is difficult to predict the characteristics of land-use changes very accurately by only using a single model. Therefore, many scholars combined the quantitative spatial coupling prediction models to simulate the temporal and spatial changes of regional land-use types after 2010. The CA-Markov model is the combination of CA and Markov models. It can not only accurately predict the quantity of land-use types but also effectively simulate the spatial distribution of land-use types, which has great practicability [16,17,18,19]. Although some scholars applied other models, such as the Future Land Use Simulation (FLUS) model [20], Artificial Neural Network (ANN)-CA model [21,22], and Land Change Modeler (LCM) model [23], the CA-Markov model is more popular in predicting the future land-use patterns than other models [24,25].

Model accuracy verification is a necessary step to determine whether the model can be applied further. The accuracy verification of models usually requires the comparison and analysis of model simulation results with actual observations. At present, the most common method to verify the accuracy of land-use simulation models is to compare the land information between different classes of two maps by comparing the simulated map with another reference map and calculating the relevant formula [26]. Some scholars applied quantitative accuracy verification by calculating the quantity errors of a certain land-use type and spatial accuracy verification by calculating the Kappa index [27,28]. However, Pontius (2011) and other relevant scholars believe the above methods were invalid and misleading in the accuracy assessment, and they even found serious defects in the land-use model [24]. Comparatively speaking, the Figure of Merit (FOM) method is better. The FOM method is a three-map comparison method in which the quantity error and space allocation error in the simulation map are judged by the superposition and comparison analysis of the simulated change map and referenced change map [29,30,31]. Therefore, the highlights of this study are to combine GIS with the widely used model, the CA-Markov model, to predict the future land-use changes and apply the FOM method to verify the accuracy of the CA-Markov model.

Maize is one of the major crops in Northeast China, and its cultivated area in Northeast China accounts for about 30% of that in China [32]. Therefore, the objectives of this study were the following: (1) use the FOM method to verify the accuracy of the CA-Markov model, employ the CA-Markov model to simulate land use in Northeast China in 2050, and analyze the land use changes during 2015–2050; (2) predict the maize potential production and yields in 2050 using the GAEZ model; and (3) explore the spatio-temporal changes of maize potential production and yields during 2015–2050. Simulating future land use, especially cropland changes, and then predicting future crop potential yields are of great significance so as to provide reference for policy makers to develop appropriate plans to cope with future cropland changes actively and improve the ability of agricultural systems to meet the growing food demand and ensure food security.

## 2. Data and Methods

### 2.1. Study Area

**Figure 1:**
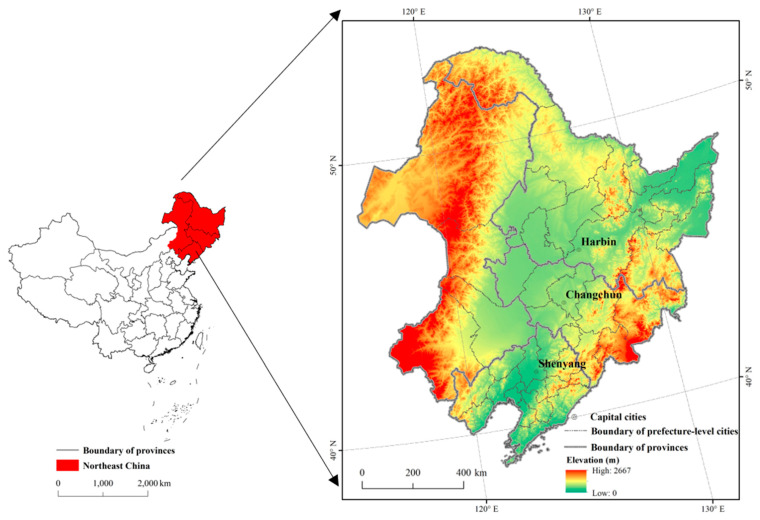
Location of the study area in China.

**Figure 2:**
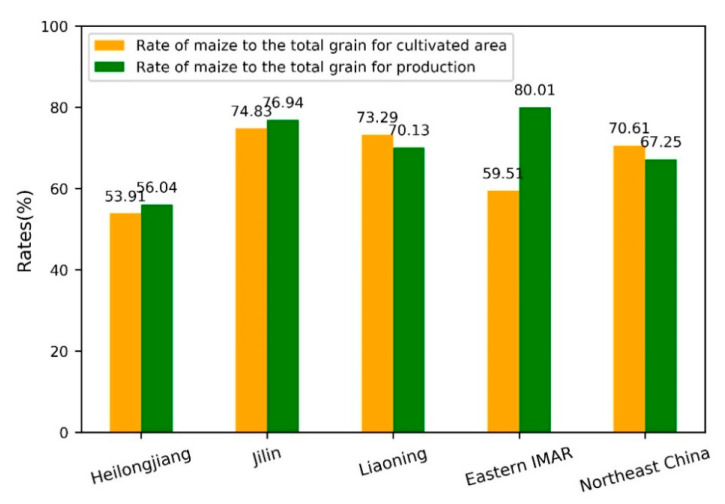
Rate of maize to the total grain for cultivated area and production in 2015.

Northeast China is located in the northeast of China’s mainland, covering 40 prefecture-level cities and four provinces including Heilongjiang, Jilin, Liaoning, and the eastern parts of the Inner Mongolia Autonomous Region (IMAR). It extends from 38°40′ N to 53°34′ N, and 115°05′ E to 135°02′ E and has the total area of about 1.24 million km^2^ (Figure 1). The Northeast China is surrounded by middle and low mountains along three directions, such as the Greater Khingan Mountains in the northwest, the Lesser Khingan Mountains in the northeast, and the Changbai Mountains in the southeast [33]. The central part is the Northeast China Plain, with Sanjiang Plain in the northeast corner, Songnen Plain in the middle, and Liaohe Plain in the south [34]. Northeast China is located in the continental monsoon climate area with mild and humid summer and bitter and long winter [35]. The annual average temperature is −5 to 10.6 °C, and the ≥10 °C annual accumulated temperature is 2200–3600 °C from north to south. The annual accumulated precipitation ranges from 1000 mm in the east to 400 mm in the west [36]. The major soil types are brown coniferous soil, dark brown forest soil, forest steppe chernozem, and meadow steppe chernozem [37]. The cropland in Northeast China accounts for 1/3 of the total area and has great agricultural production potential. The major crops planted in Northeast China include maize, soybean, rice, wheat, millet, and sorghum, among which maize is the most popular. By referring to the Statistical Yearbook in Northeast China obtained from the Resources and Environment Science and Data Center (http://www.resdc.cn/), the maize cultivated area and production account for about 70% of the total grain (including cereal, tuber, and beans) in Northeast China in 2015 (Figure 2).

### 2.2. Data Sources and Preprocessing

Land use, climate, soil, terrain, some socioeconomic statistical data (population density, Global Domestic Product (GDP), and grain cultivated area and production), as well as some other geographical data (highway, railway, cities, and villages) were needed in this study to simulate future land use by the CA-Markov model and predict maize potential yields by the GAEZ model. The detailed data sources and preprocessing have been shown as follows.

#### 2.2.1. Data for Future Land-Use Simulation

The land-use data in years 1990, 2000, and 2015 were used to simulate future land use in the CA-Markov model in this study. They were derived from the land-use database (with a mapping scale of 1:100,000) developed by the Chinese Academy of Sciences (CAS) (http://www.resdc.cn/). The database was obtained from manual visual interpretation at Landsat Thematic Mapper/Enhanced Thematic Mapper (TM/ETM) images in years 1990 and 2000, and Operational Land Imager (OLI) images in 2015. According to Liu et al.’s studies and data producers’ validation based on various geophysical datasets (e.g., soil type, Digital Elevation Model (DEM), roads and rivers, and climate) and field survey, the interpretation accuracy of the land-use database was >90%, which could satisfy the accuracy requirement of 1:100,000 mapping [38,39]. The land-use data in three years were classified using the first-grade types of the datasets, i.e., cropland, woodland, grassland, water body, built-up land, and unused land [40,41]. Then, the cropland was further divided into paddy field and dryland. The maps for 1990, 2000, and 2015 helped to capture the transition rule and historical development trends. Meanwhile, the map in 2015 was the reference map used to verify the simulation accuracy of the CA-Markov model when the maps for 1990 and 2000 were used for predicting the land use in 2015.

Land-use changes can be affected by various natural and humanity factors. Therefore, nine driving factors, including DEM, slope, aspect, distance from the highway and railway, population density, GDP, and distance from the cities and villages were considered. The DEM data with 90 m spatial resolution were derived from the Shuttle Radar Topography Mission (SRTM) C-band data. Furthermore, the DEM data were processed into slope and aspect data. The highway, railway, population density, and GDP data were obtained from the Resources and Environment Science and Data Center (RESDC) (http://www.resdc.cn/). The highway and railway data were processed to the distance data using the IDRISI software (Clark Labs, Worcester, MA, USA). Similarly, the cities and villages data were extracted from the land-use data in 2000 and 2015, and further processed to the distance data.

#### 2.2.2. Data for Maize Potential Yields Prediction

As a critical natural influencing factor of maize growth, the climate data need to be input to the GAEZ model in this study. The data were seven kinds of climate variables in 2015, including monthly average maximum and minimum temperature, cumulative precipitation, cumulative net solar radiation, average relative humidity, average wind speed at 2 m height, and wet day frequency (the number of days on which the precipitation exceeds 0.2 mm). The original observations for the above seven climate variables came from monthly observations of 99 meteorological stations distributed throughout the study area at a wide range of elevations, which were obtained from the National Meteorological Information Center (http://www.nmic.cn/). By using the ANUSPLIN software (Australian National University, Canberra, Australia), the original climate observations were interpolated to continuous grid surfaces with 10 km spatial resolution based on the DEM of Northeast China [42,43].

The soil data of the study area with 1 km spatial resolution were derived from 1:1,000,000 scale Soil Map of China provided by the Institute of Soil Science, CAS, which were based on the Harmonized World Soil Database (HWSD) developed by the Food and Agriculture and Organization of the United States (FAO) and the International Institute for Applied Systems Analysis (IIASA) [44]. The data were the combination of soil spatial distribution and soil profile attributes such as organic carbon content, Pondus Hydrogenii (PH), cation exchange capacity, soil texture, and soil water-holding capacity.

The terrain data input to the GAEZ model included DEM, slope, and aspect data. They were all resampled to a 1 km spatial resolution grid.

The dryland data in 2015 were extracted from the land-use data and further processed to dryland ratio grid with 1 km spatial resolution as the input data for the GAEZ model.

Irrigation data, grain cultivated area, and production data in 2015 were also needed. Both of them were obtained from the Statistical Yearbook of Northeast China in 2015 obtained from the Resources and Environment Science and Data Center (http://www.resdc.cn/) and took the prefecture-level city in Northeast China as the statistical level. The irrigation data were processed to the irrigation ratio grid data with 1 km spatial resolution representing the ratio of irrigated area to the total area in each grid cell.

### 2.3. Methods

#### 2.3.1. Future Land-Use Simulation

Simulating future land use involves measuring land-use changes between time *t*1 and *t*2, and then extrapolating the changes into the future [45]. The CA-Markov model, which is the combination of the Markov model and Cellular Automata (CA) model, was used to simulate future land use in this study.

The Markov model is a traditional theory method based on the formation of Markov random process systems for the prediction [46]. It is often used to predict geographical events with non-aftereffect property [47,48]. In the research of land-use changes, the Markov model analyzes the probability of land-use changes over time by developing a transition probability matrix and predicting the quantity of land-use types for some time according to the transition probability matrix in the previous period. Therefore, the state of land-use types at time *t* + 1 depends on the state of land-use types at time *t*. The state of land-use types at time *t* + 1 can be calculated by the following formula [47,48,49,50]:

(1) $$S_{t+1}=P_{ij}\times S_{t},$$

where $S_{t+1}$, $S_{t}$ are the states of land-use types at time *t* + 1 and *t*, while $P_{ij}$ is the transition probability matrix between land-use types *i* and *j*. $P_{ij}$ is calculated as follows [49]:

(2) $$P_{ij}=\frac{A_{ij}}{\sum_{j=1}^{n}A_{ij}}\times 100\%$$

where $A_{ij}$ is the area or proportion of land-use type *i* converted to *j*, and n is the number of land-use types.

The CA model is a complex dynamic model discrete in time, space, and state. The model has been widely used in the simulation of land-use changes [12,13]. The basic elements of the model mainly include cellular space and cell size, state collection, state transition rule, and neighborhood scope, etc. The CA model can be expressed by the following formula [49]:

(3) $$S_{t+1}=f\left(S_{t},\;N\right),$$

where $S_{t+1}$, $S_{t}$ are also the states at time *t* + 1 or *t*, *N* is the cellular field, and f is the transition rule of cellular states in local space.

In the CA-Markov model, the Markov process uses the transition probability matrix of land-use states to reflect the transition probability within the specific land unit, which can be used to explain the quantitative relationship in the process of land-use changes. The spatial parameters are weak, and the various types of land-use changes in the spatial extents are unknown [51]. The CA process can explain the spatial variables, plot interaction, and driving mechanism in the process of land-use changes. Therefore, the CA-Markov model incorporates the theories of Markov and CA, and it is a very efficient tool for imitating complex spatial processes based on simple decision rules [52]. It can achieve better simulation for land-use changes in quantity and space [53,54,55].

**Figure 3:**
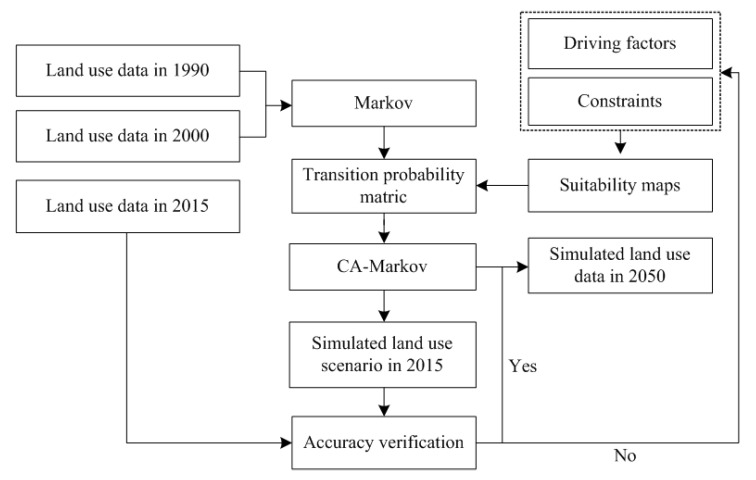
Framework of land-use simulation.

In this study, the IDRISI Selva software v.17 (Clark Labs, Worcester, the United States) was used for land-use simulation. Firstly, the CA-Markov model was used to simulate land use in 2015. The transition probability matrix was obtained from land-use data in 1990 and 2000. The accuracy of the CA-Markov model was verified by comparing the simulated land use in 2015 to the land-use data of visual interpretation in 2015. Then, the CA-Markov model was used to simulate land use in 2050 based on the transition probability matrix between land-use types in 2000 and 2015. The framework of land-use simulation is summarized in Figure 3.

The following is the detailed process of land-use simulation by the CA-Markov model. Take land-use simulation in 2015 as an example

(1) Land-use transition probability matrix during 1990–2000

First, this study used the Markov module in IDRISI to calculate the transition matrixes during 1990–2000, including the transition area matrix and probability matrix. In the Markov module, the normal allocation proportional error was set to 0.15. By superimposing the land-use maps of 1990 and 2000, the transition area matrix and probability matrix were obtained, in which the transition area matrix was used as a parameter to predict the land-use changes during 2000–2015.

(2) Transition suitability maps of land-use types

Transition suitability maps of land-use types are the probability maps of the transition of one land-use type into another, which can be generated by the Multi-Criterion Evaluation (MCE) module in IDRISI. The MCE module can be further divided into constraints and driving factors. Constraints are used to limit the range of land-use type transitions, which are expressed in the form of Boolean and standardized to binary images. Driving factors are the criterion that enhance or detract from the suitability of land-use type transition. Driving factors are standardized to a scale of 0–255 by selecting suitable function shapes (including monotonically increasing, monotonically decreasing, and symmetric), functions types (including sigmoidal, J-shaped, and linear) and control points (including a, b, c, and d) in the Fuzzy module of IDRISI.

Generally speaking, land-use changes can be affected by various natural and humanity factors. Therefore, when simulating land use in 2015, nine driving factors including elevation, slope, aspect, distance from the highway and railway, population density in 2000, GDP in 2000, and distance from the cities and villages in 2000 were selected as the driving factors. The water distribution map in 2000 was considered as the constraint, indicating that water could not be converted to some other land-use types. Table 1 showed the constraints and function shapes, types, and values of control points of various driving factors when simulating land use in 2015. By the Fuzzy module, the values of various driving factors were stretched to continuous values between 0 and 255.

Next, we used the Analytical Hierarchy Process (AHP) to determine the weights of nine driving factors on land-use types according to the importance of each factor to land-use type transition. In this step, five experts set the weights by comparing driving factors pairwise. Table 2 showed the weights of the driving factors. The consistency ratios were 0.01 or 0.02. Since we believed that the transition of water body was not affected by the above driving forces, we did not make the suitability map of water body. Meanwhile, we used a Weighted Linear Combination (WLC) to achieve a balance between positive and negative qualities in assessing suitability, so the No Ordered Weighted Averaging (OWA) method was used in this step. Finally, the transition suitability maps of land-use types were obtained.

(3) Land-use simulation in 2015

In the last step, the CA-Markov module was employed to simulate the land use in 2015 based on the land-use data of visual interpretation in 2000, transition probability matrix from 2000 to 2015, and transition suitability maps.

In this study, we used the Figure of Merit (FOM) method to test the consistency between the simulated land use in 2015 and the land-use data of visual interpretation in 2015. The referenced change map can be obtained by superimposing the reference maps at the start time *t*1 and the end time *t*2, and the simulated change map is obtained by superimposing the reference map at *t*1 and simulation map at *t*2. By comparing the referenced change map with the simulated change map, the accuracy of the model can be determined by five indicators: Misses (M), Hits (H), Wrong Hits (W), Null successes (N), and False Alarms (F). M is the area proportion of error due to reference change simulated as persistence; H refers to the area proportion of correct due to reference change simulated as change; W refers to the area proportion of error due to reference change simulated as change to the wrong category; N is the area proportion of correct due to reference persistence simulated as persistence; and F refers to the area proportion of error due to reference persistence simulated as change [56,57]. Based on these five indicators, a series of validation indicators are proposed, such as referenced change (RC), simulated change (SC), error due to quantity (EQ), error due to allocation (EA), total error (TE), and FOM. The formulas are as follows

(4) M+H+F+W+N=100%

(5) RC=M+H

(6) SC=H+F

(7) EQ=|SC−RC|=|F−M|

(8) EA=2×Minimum(F,M)

(9) TE=EQ+EA

(10) FOM=H/(H+W+M+F)×100%.

EQ refers to the error caused by the model’s imprecise simulation of the net change quantity, which is not caused by space allocation. EA refers to the error caused by space allocation. The range of FOM is from 0% to 100%, in which 0% means that there is no overlap between referenced change map and simulated change map, while 100% means that there is complete overlap between referenced change map and simulated change map. In this study, we calculated H, W, M, F, N, RC, SC, EQ, EA, TE, and FOM by superimposing the referenced and simulated change maps from 2000 to 2015, so as to verify the accuracy of the CA-Markov model.

#### 2.3.2. Analysis of Future Land-Use Changes

Then, we simulated land use in Northeast China in 2050 by the CA-Markov model and analyzed the land-use changes in Northeast China between 2015 and 2050. By analyzing the transition probability matrix, the characteristics of the mutual transition of land-use types were discussed.

#### 2.3.3. Prediction of Maize Potential Yields

The AEZ model was developed by the Food and Agricultural Organization (FAO) of the United Nations and the International Institute for Applied System (IIASA) primitively, and the Global AEZ model (GAEZ) model has been widely used in predicting crop potential yields recently [58,59,60]. It is a simple and robust crop model and provides standardized crop modeling and an environmental matching procedure to calculate crop potential yields based on photosynthetic potential, light and temperature potential, climate production potential, and land production potential step by step under assumed input and management levels. Several articles have given a detailed description of the calculation procedures of the GAEZ model [61,62]. This study extracted a dryland distribution map from the simulated land use in 2050 and then predicted maize potential yields in 2050 using the GAEZ model under the simulated dryland. In order to predict maize potential yields under future land use, the prediction ensured that the climate, soil, terrain, and irrigation ratio maintained the status of 2015.

Model accuracy verification is a necessary step to determine whether the model can be applied further. Previous studies have verified the accuracy of the GAEZ model by setting up a regression relationship between the crop actual yields and the crop potential yields simulated by the GAEZ model [61,62,63]. Results showed that the coefficients of determination of regression equations were all more than 0.75, showing that the GAEZ model had an appropriate capability to simulate crop potential yields.

#### 2.3.4. Analysis of Maize Potential Yields Changes

The temporal and spatial changes of maize potential yields during 2015–2050 were obtained by comparing the maize potential yields in 2015 and 2050. Temporally, this study calculated the quantity changes of maize potential production and average potential yields during 2015–2050 in each province and the whole Northeast China. Spatially, this study analyzed the spatial distribution characteristics of maize potential yields changes and the changes of maize potential production and average potential yields in prefecture-level cities.

## 3. Results

### 3.1. Accuracy Verification of the CA-Markov Model

**Figure 4:**
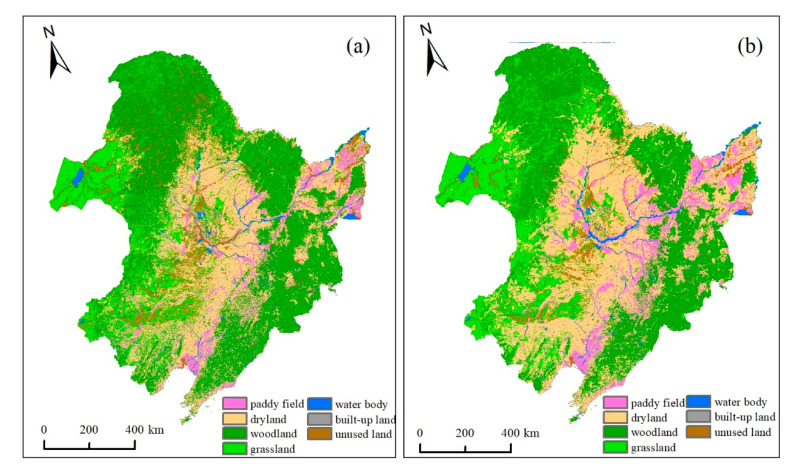
Land-use data of visual interpretation (**a**) and simulated land use (**b**) in 2015.

First, this study used ArcGIS, ENVI, and IDRISI Selva software and the CA-Markov model to simulate land use in Northeast China in 2015 based on the land-use data in 1990 and 2000. Figure 4 showed the land-use data of visual interpretation and simulated land use in 2015. We further calculated the transition area of various land-use types from 2000 and 2015 (Table 3). The transition trend of seven land-use types in the referenced and simulated change maps were similar. The total transition area of land-use types was 35.03 million ha in the referenced change map and 26.52 million ha in the simulated change map.

Next, we superimposed the referenced and simulated change maps from 2000 to 2015 and calculated various indicators. Among these, the H, W, M, F, and N were 33.71%, 8.80%, 21.34%, 3.49%, and 32.66% respectively, so the RC was 55.05% and SC was 37.20%. EQ was 17.85%, and EA was 6.98%, so the TE was 33.63%, indicating that there were more errors in quantity than in space allocation in this simulation by the CA-Markov model. The FOM was 50.06%.

The FOM in this simulation was higher than that in other simulations [64,65], indicating that the accuracy of the CA-Markov model was good. However, if we had examined only the single FOM metric, then we would not be able to have the insights that we had from interpretation of Misses, Hits, Wrong Hits, and False Alarms [56]. In other words, we may not be able to know whether inconsistency derives from quantity or allocation. Therefore, other indicators should not be neglected. The EQ was greater than EA, showing that the inconsistency between simulated land use in 2015 and land-use data of visual interpretation were derived more from the Markov algorithm than from the CA model.

### 3.2. Land-Use Changes During 2015–2050

After verifying the accuracy of the CA-Markov model, this study simulated land use in Northeast China in 2050 based on the land-use data in the years 2000 and 2015. Next, according to the spatial overlay analysis in ArcGIS, from 2015 to 2050, there were apparent changes among the land-use types. The area and proportion changes are shown in Table 4. The main land-use types in Northeast China are woodland and dryland, which account for 40% and 25% of the total respectively. Grassland and unused land had large area and proportions, while the area of water body and unused land was small.

From 2015 to 2050, the expansion of unused land and reduction of woodland were sharp. The area and proportion of unused land increased by 3.61 million ha and 2.92% respectively, while woodland decreased by 5.14 million ha and 4.15%. In addition, paddy field and built-up land increased obviously over the 35 years, with the large-scale decline of grassland. The results showed that the ecological environment in Northeast China may be deteriorated over the 35 years, and the cropland reclamation and built-up land expansion would be very drastic.

**Figure 5:**
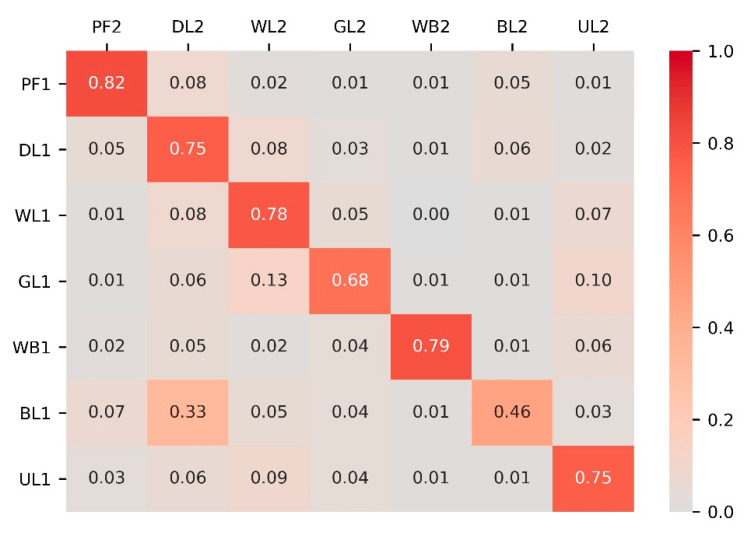
Transition probability matrix of land-use types in Northeast China during 2015–2050. Note: The axes letters have the same meanings as Table 3. The number 1 represents 2015, and 2 represents 2050.

So, how did land-use types transfer to the others? Figure 5 was the heat map showing the transition probability matrix of seven land-use types in Northeast China during 2015–2050. The conversion of paddy field was slow compared with the other land-use types; 13% was converted to dryland. Dryland was converted to woodland and built-up land, and the proportions were 8% and 6%, respectively. A large area of woodland was converted to dryland (8%) and unused land (7%). Grassland was mainly converted into woodland (13%) and unused land (10%). Since the water body in 2015 was used as a constraint to simulate the land-use scenario in 2050, there were few changes between the water body and other types. A large area of built-up land was converted to dryland (33%). As for unused land, 9% was converted to woodland. Combined with the area and proportion changes of land-use types in Table 3, the land-use changes in Northeast China from 2015 to 2050 were mainly the conversion of forest and grass to unused land. The conversion of dryland to paddy field and built-up land was quite dramatic. Therefore, in the coming decades, more ecological and environmental protection policies should be formulated in Northeast China to prevent the mass loss of woodland and grassland and the vast increase of unused land. In addition, the acceleration of economic development and urbanization process will lead to the continuous expansion of built-up land, but it should not be at the expense of high-quality cropland resources. Farmland resources protection is still very urgent.

### 3.3. Maize Potential Yields in 2050

**Figure 6:**
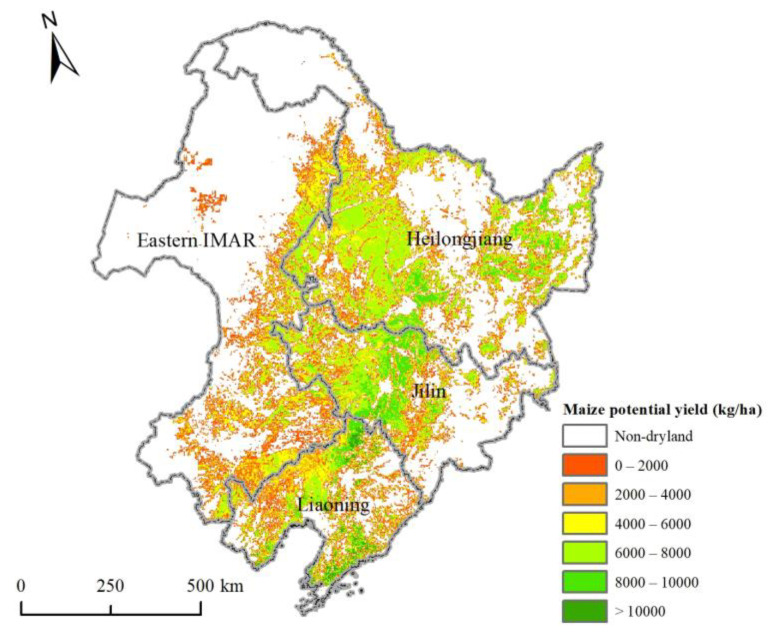
Spatial distribution of maize potential yields in Northeast China in 2050.

Maize potential yields in 2050 were predicted using the GAEZ model discussed in Section 2.3.3, as well as the seven kinds of climate variables, DEM, soil, dryland, and irrigation data mentioned in Section 2.2.2. The distribution map of maize potential yields in Northeast China in 2050 is shown in Figure 6. In the whole study area, the total potential production was 218.09 million tonnes. As the crop production is equal to yield times crop planting area, and the simulated dryland area was 31.70 million ha, the average maize potential yield was 6880.59 kg/ha. The maize potential yields in the center region were more than 8000 kg/ha, which is much higher than that in the other regions. The potential yields even exceeded 10,000 kg/ha in some areas. In some west and southeastern regions, the yields were less than 4000 kg/ha.

As for the four provinces, Heilongjiang had the highest maize potential production (92.87 million tonnes), which was followed by Jilin (50.22 million tonnes), Liaoning (39.49 million tonnes), and Eastern IMAR (36.64 million tonnes). The main reason why the production of Heilongjiang was much higher than that of other provinces was the large area of dryland. The average maize potential yields in three provinces exceeded 7000 kg/ha, which were Liaoning (7866.53 kg/ha), Jilin (7440.00 kg/ha), and Heilongjiang (7014.35 kg/ha). These regions had fertile, contiguous plains with plenty of rainfall and solar radiation, which is suitable for maize growth. Eastern IMAR had the lowest average yield (5485.03 kg/ha), which was mainly due to infertile soil and insufficient light and rainfall.

**Figure 7:**
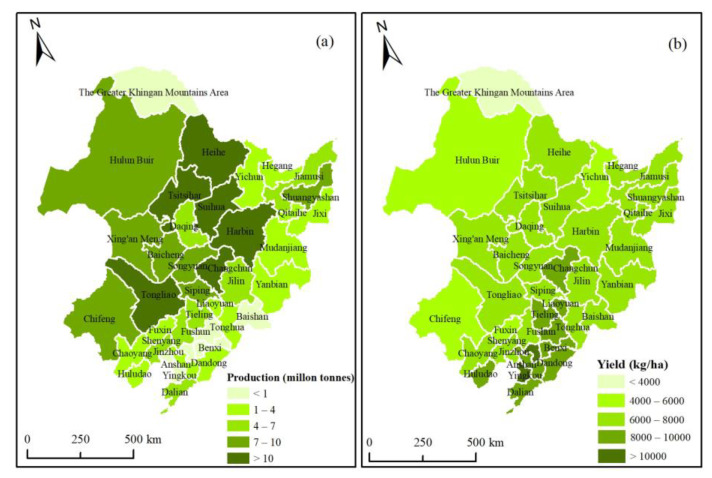
Maize potential production (**a**) and yields (**b**) in prefecture-level cities in 2050.

By further taking prefecture-level city as the scale for analysis, the cities with high maize potential production (more than 7 million tonnes) in 2050 in Northeast China were mainly distributed in the western and central regions, including Eastern IMAR, four cities in western Heilongjiang, and four cities in Jilin (Figure 7a). Six cities even had the potential production of more than 10 million tonnes. The maize potential production in eastern and southern regions, such as western Heilongjiang and Jilin, and all cities in Liaoning was less than 7 million tonnes. The main reason was the cities in western and central regions had rich dryland resources—far more than the cities in eastern and southern regions.

However, the distribution of maize potential yields in cities was not the same as that of production (Figure 7b). The cities with high maize potential yields (more than 8000 kg/ha) in 2050 were mainly distributed in the southern regions, including Changchun and Liaoyuan in central Jilin, and nine cities in Liaoning. Most cities in Northeast China had the potential yields of between 6000 and 8000 kg/ha. Hulun Buir and Chifeng in Eastern IMAR had the yields of 4000–6000 kg/ha, and the Greater Khingan Mountains Area even had the yield of less than 4000 kg/ha. Therefore, in the southern regions, the climate resources were more abundant and the soil was more fertile, which were suitable for maize growth; thus, the maize potential yields were higher. Although the western and central regions had a large area of dryland, the maize potential yields were limited by unfavorable climate, terrain, and soil quality.

### 3.4. Temporal and Spatial Changes of Maize Potential Yields

The spatio-temporal changes of maize potential yields from 2015 to 2050 were obtained according to the spatial overlay analysis in ArcGIS, and there were apparent changes over the 35 years.

#### 3.4.1. Temporal Changes of Maize Potential Yields during 2015–2050

**Figure 8:**
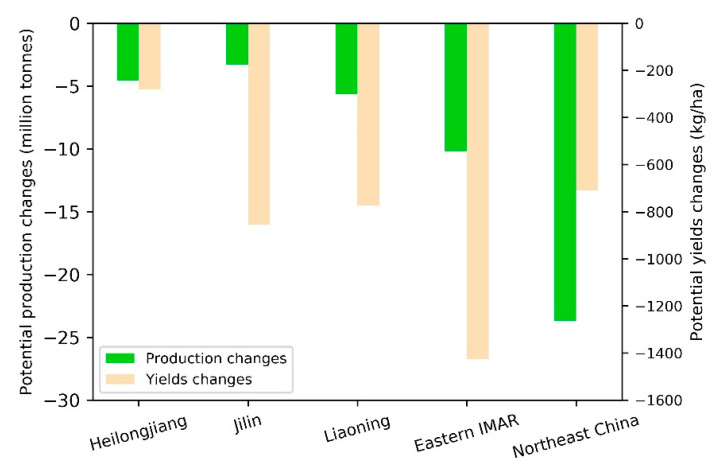
Maize potential production and yields changes during 2015–2050.

Figure 8 showed the total maize potential production and average potential yields changes in Northeast China and the four provinces during 2015–2050. It was shown that the maize potential production and average yield in Northeast China declined under the impact of land-use changes over the 35 years. The total maize potential production decreased by about 23 million tonnes, and the average potential yield decreased by around 700 kg/ha. As for the four provinces, the potential production and average yield in Eastern IMAR decreased the most, which were more than 10 million tonnes and 1400 kg/ha, respectively. Jilin had the least reduction in maize potential production (about 3 million tonnes). The average potential yield in Heilongjiang reduced by less than 300 kg/ha, which was the least. The decrease of maize potential production in the whole study area and the four provinces was mainly due to the reduction of dryland, while the decrease of average potential yields may be due to the lower dryland quality in 2050 than that in 2015.

#### 3.4.2. Spatial Changes of Maize Potential Yields during 2015–2050

**Figure 9:**
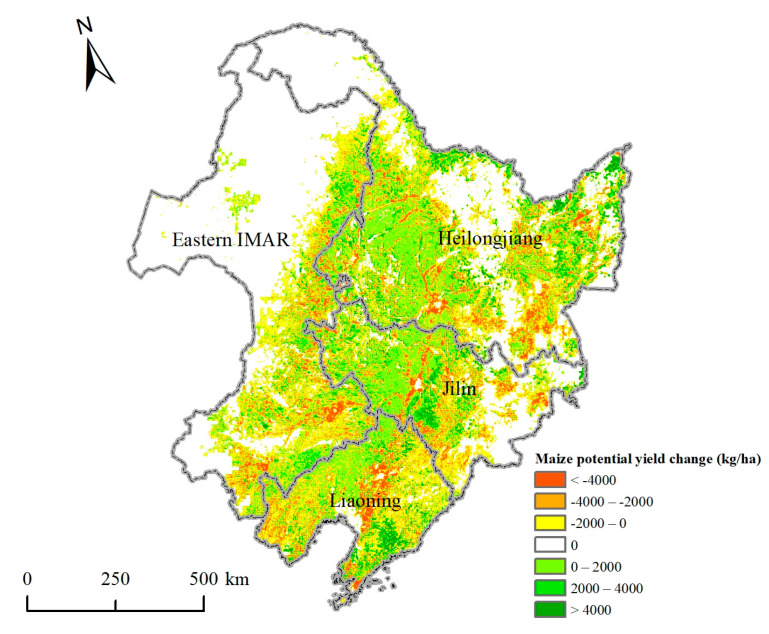
Spatial changes of maize potential yields in Northeast China during 2015–2050.

Figure 9 was the spatial changes map of maize potential yields in Northeast China during 2015–2050. Although the average potential yields in Northeast China and the four provinces decreased, the potential yields in some regions still rose over the 35 years. The maize potential yields increased by less than 2000 kg/ha in about 1/3 of the dryland, which was intensively distributed in the plain areas. In central Jilin and southern Liaoning, the maize potential yields even increased by more than 4000 kg/ha. Meanwhile, it was easy to find that the yields reduced by less than 2000 kg/ha in nearly 1/3 of the dryland, which were widely distributed in Eastern IMAR, central Jilin province, and eastern Liaoning province. In some regions of Eastern IMAR, southern Heilongjiang, eastern Jilin, and central Liaoning, the yields decreased by more than 4000 kg/ha.

**Figure 10:**
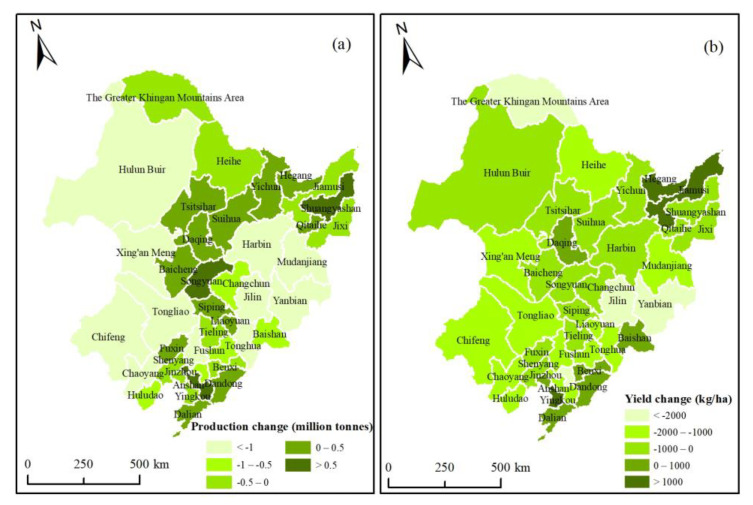
Maize potential production changes (**a**) and yield changes (**b**) in prefecture-level cities during 2015–2050.

Figure 10 showed the changes of maize potential production and yields in prefecture-level cities of Northeast China over the 35 years. Although the maize potential production in the whole Northeast China and the four provinces declined overall, it increased in some cities (Figure 10a). In the Sanjiang Plain, central and southern regions, the potential production increased. The production in Shuangyashan, Songyuan, and Anshan even increased by more than 0.5 million tonnes. In most of the cities of the western and eastern regions, the maize potential production reduced by more than 1 million tonnes. Dryland changes were the important reason for maize potential production changes. The production changes were mainly due to the dryland shrinkage in western and eastern regions and the dryland expansion in the plain regions. 

The changes of maize potential yields indicated the dryland quality changes caused by the conversion process of land-use types between 2015 and 2050. The maize potential yields increased in only nine cities in Northeast China, mainly in Hegang and Jiamusi within the Sanjiang Plain, and several cities in the southeastern regions. Hulunbuir, as well as most cities in Heilongjiang and western Jilin, saw the maize potential yields reduced by less than 1000 kg/ha, while the other three cities in Eastern IMAR, and most cities in Liaoning saw the yields decreased by 1000–2000 kg/ha. 

## 4. Discussion

### 4.1. Kappa Index Method

Some studies supposed the Kappa index could test the spatial consistency between the simulated results and observed data. The Kappa index can be calculated by the following formula [27]:

(11) $$\mathrm{Kappa}=\frac{P_{o}-P_{c}}{P_{p}-P_{c}}$$

where $P_{o}$ represents the ratio of grid cells to be simulated correctly, $P_{c}$ represents the ratio of grid cells to be simulated correctly in the random case, and $P_{p}$ represents the ratio of grid cells correctly in the ideal state, which is 100%. The range of Kappa index is (–1–1). Some scholars believe that if 0.75 ≤ Kappa ≤ 1, the simulation results and observed data are in a high level of spatial agreement; if 0.50 ≤ Kappa < 0.75, it has a medium level of spatial agreement; and if Kappa < 0.5, it has a rare spatial agreement [27,28].

In this study, we also used the Kappa index method synchronously so as to compare with the FOM method. We used the Crosstab module in IDRISI to calculate the level of agreement between the simulated land-use scenario in 2015 and the land-use data of visual interpretation in 2015. The kappa indexes between the simulated land-use scenario and the land-use data of visual interpretation in 2015 are shown in Table 5, including the indexes of the entire map and six kinds of land-use types. The Kappa index was >0.75 between the simulated land use and the land-use data of visual interpretation. Kappa indexes of different land-use types were woodland > dryland > water body > built-up land > grassland > paddy field > unused land. The Kappa index of woodland was up to 0.85. However, the Kappa indexes of unused land and dryland were only 0.57 and 0.66. The main reason was that the land-use changes after 2000 were more affected by human disturbance, especially the deforestation of woodland for cropland, and the destruction of woodland and grassland, resulting in the dramatic increase of the unused land such as saline-alkali land and sandy land. Sometimes, the impact of human activities on land-use changes can not be accurately simulated by the CA-Markov model.

By comparing the Kappa index method with FOM method, it can be found that the FOM method is more suitable for evaluating the accuracy of the CA-Markov model. The main reason was as follows. The CA-Markov model allocated the gain of each category around patches of the category at the start time, which caused long winding patches of simulation change. The shapes of the patches of simulation change do not match the compact and isolated patches of the reference change. The Kappa index method may sometimes lead to misestimates of spatial consistency. It may be more suitable for simply comparing the simulation accuracy of different land-use types. From the Kappa indexes of each land-use type above, we can believe that the simulation of woodland was more accurate than that of the other land-use types. However, the accuracy of the whole simulated map in 2015 cannot be considered as high according to the overall Kappa index of 0.77. Therefore, the Kappa index is not an appropriate choice in the image construction process.

By comparison, the FOM method is better. The FOM method can not only evaluate the accuracy of simulation but also distinguish the quantity error and space allocation error. This method is more practical than the Kappa index method in evaluating the consistency of pixels between the simulated map and the reference map.

### 4.2. More Work Needed in Considering Driving Factors and Constraints

Since land-use changes can be affected by various natural and humanity factors, this study selected nine driving factors to simulate future land use. Meanwhile, the water distribution maps in 2015 and 2050 were also taken as the constraints. However, the consideration of driving factors and constraints remains to be further optimized. First, some climate factors, such as precipitation, may affect the distribution of unused land. Accumulated temperature may affect the distribution of cropland. However, it is difficult to accurately evaluate the impact of climate factors on land-use changes in the study area, so climate factors were not taken as the driving factors in this study. Second, due to the policy of prohibiting the occupation of some ecological land and the red line of cropland in China, some nature reserves and basic farmland should also be considered as constraints. However, because it is difficult to obtain relevant data, this study only considered water distribution maps as constraints. Some subjective human activities may have a great impact on land-use changes. For example, the site selection of built-up land may be greatly affected by human subjective preferences. It is difficult to use the CA-Markov model to simulate the influence of some human subjective activities. Therefore, an insufficient selection of driving factors and constraints as well as the unpredictability of some subjective factors may result in inaccurate simulation results. Therefore, more work needs to be done to consider driving factors and constraints in the future study.

### 4.3. Impact of Accuracy Verification of Land-Use Simulations on Maize Potential Yields Prediction

In this study, we used the FOM method to verify the accuracy of the land-use simulation in 2015 and found that there were more errors in quantity than in space allocation by the CA-Markov model. Similarly, when we used the CA-Markov model to simulate land use in 2050, there was a great possibility that errors in quantity were more than that in space allocation because the same driving factors and constraints were implied in the two land-use simulations. Therefore, this also affected the prediction results of maize potential yields in 2050. It can be inferred that errors in the quantity of maize potential yields in 2050 may be more than that in the spatial distribution. First, this illustrates that as described in Section 4.2, future work should try to comprehensively select driving factors and constraints to reduce the quantity and space errors of land-use simulations, so as to further reduce the errors of crop potential yields prediction. Second, it is further shown that the FOM method is superior to the Kappa index method. If we use the Kappa index method, we cannot know whether the errors in the prediction of maize potential yields in 2050 are more due to quantitative or spatial allocation.

## 5. Conclusions

In the present study, the CA-Markov model was employed to simulate land-use changes in Northeast China during 2015–2050. Then, the maize potential yields in 2050 were predicted by the GAEZ model, and the maize potential yields changes were analyzed. Finally, this study put forward some effective suggestions for the future food security in Northeast China according to the results above.

According to the land-use simulation in 2050, the ecological environment in Northeast China showed a trend of deterioration from 2015 to 2050. The area of woodland and grassland decreased dramatically. Dryland also shrank by 0.32 million ha. The land-use changes from 2015 to 2050 were mainly the conversion of forest and grass to unused land, and the conversion of dryland to paddy field and built-up land.

In 2050, the total maize potential production and average potential yield were 218.09 million tonnes and 6880.59 kg/ha in Northeast China. In the center region, the maize potential yields were much higher than that in the other regions, which were more than 8000 kg/ha. As for the maize potential yields and production changes, from 2015–2050, the total maize potential production decreased by about 23 million tonnes due to the reduction of dryland area, and the average potential yield decreased by about 700 kg/ha due to the decline of dryland quality. The maize potential yields decreased in most regions, but they increased by less than 2000 kg/ha in about one-third of the dryland, which was intensively distributed in the plain areas.

In view of the above results, the following suggestions were proposed. In the coming decades, more ecological and environmental protection policies should be formulated in Northeast China to prevent the mass loss of woodland and grassland and the vast increase of unused land. In addition, in order to prevent the expansion of built-up land at the expense of high-quality farmland, cropland protection is still very urgent. The important basis of crop production is to ensure the quantity and quality of cropland resources. Therefore, under the premise of farmland protection, improving the quality of cultivated land by increasing organic fertilizers, applying biological fertilizers, carrying out reclamation of degraded farmland, as well as strengthening soil and water conservation will be the top priority. This will be the best way to increase crop yields and ensure food security.

## Figures and Tables

**Table 1:** Function information of driving factors and constraints of land-use type transition.

Factors		Cropland	Woodland	Grassland	Water Body	Built-Up Land	Unused Land
Constrains		Water	Water	Water		Water	
Driving factors	DEM (m)	monotonically decreasing; sigmoidal; c = 100, d = 900	monotonically increasing; sigmoidal; a = 100, b = 2000	monotonically decreasing; sigmoidal; c = 50, d = 1000		monotonically decreasing; sigmoidal; c = 50, d = 300	monotonically decreasing; sigmoidal; c = 50, d = 300
	Slope (°)	monotonically decreasing; J-shaped; c = 2, d = 15	monotonically increasing; J-shaped; a = 2, b = 25	monotonically decreasing; J-shaped; c = 10, d = 30		monotonically decreasing; J-shaped; c = 2, d = 15	monotonically decreasing; J-shaped; c = 5, d = 15
	Aspect (°)	symmetric; sigmoidal; a = 10, b = 180, c = 210, d = 350	symmetric; sigmoidal; a = 10, b = 180, c = 210, d = 350	symmetric; sigmoidal; a = 10,b = 180, c = 210, d = 350		symmetric; sigmoidal; a = 10,b = 180, c = 210, d = 350	symmetric; sigmoidal; a = 10, b = 180, c = 210, d = 350
	Distance from the highway (m)	monotonically decreasing; sigmoidal; c = 2000, d = 50,000	monotonically decreasing; sigmoidal; c = 500, d = 100,000	monotonically increasing; sigmoidal; a = 2000, b = 100,000		monotonically decreasing; sigmoidal; c = 500, d = 50,000	monotonically increasing; sigmoidal; a = 500, b = 100,000
	Distance from the railway (m)	monotonically decreasing; sigmoidal; c = 4000, d = 100,000	monotonically decreasing; sigmoidal; c = 500, d = 100,000	monotonically increasing; sigmoidal; a = 2000, b = 100,000		monotonically decreasing; sigmoidal; c = 500, d = 50,000	monotonically increasing; sigmoidal; a = 500, b = 100,000
	Population density (people/km^2^)	monotonically decreasing; sigmoidal; c = 500, d = 5000	monotonically decreasing; sigmoidal; c = 500, d = 3000	monotonically decreasing; sigmoidal; c = 500, d = 3000		monotonically increasing; sigmoidal; a = 100, b = 3000	monotonically decreasing; sigmoidal; c = 100, d = 3000
	GDP (10^4^ yuan/km^2^)	monotonically decreasing; sigmoidal; c = 500, d = 2000	monotonically decreasing; sigmoidal; c = 500, d = 5000	monotonically decreasing; sigmoidal; c = 500, d = 5000		monotonically increasing; sigmoidal; a = 100, b = 5000	monotonically decreasing; sigmoidal; c = 500, d = 3000
	Distance from the cities (m)	monotonically increasing; sigmoidal; a = 1000, b = 30,000	monotonically increasing; sigmoidal; a = 1000, b = 30,000	monotonically increasing; sigmoidal; a = 1000, b = 30,000			monotonically increasing; sigmoidal; a = 100, b = 5000
	Distance from the villages (m)	monotonically decreasing; sigmoidal; c = 5000, d = 30,000	monotonically increasing; sigmoidal; a = 1000, b = 20,000	monotonically increasing; sigmoidal; a = 1000, b = 30,000			monotonically increasing; sigmoidal; a = 1000, b = 60,000

Note: For each of the monotonic functions, only two control points (“a” and “b”, or “c” and “d”) are needed, while the symmetric function requires four control points (“a”, “b”, “c”, and “d”). “a” is the value at which suitability begins to rise above 0, “b” is the value at which suitability attains the value 255, “c” is the value at which suitability begins to fall below 255, and “d” is the value at which suitability attains the value 0.

**Table 2:** Weights of the driving factors on land-use types.

Driving Factors	DEM	Slope	Aspect	Distance from the Highway	Distance from the Railway	Population Density	GDP	Distance from the Cities	Distance from the Villages
Cropland	0.1519	0.1894	0.1894	0.0919	0.0919	0.0508	0.0508	0.0919	0.0919
Woodland	0.1878	0.1878	0.1292	0.1091	0.1091	0.0466	0.0466	0.0919	0.0919
Grassland	0.1878	0.1878	0.1292	0.1091	0.1091	0.0466	0.0466	0.0919	0.0919
Water body									
Built-up land	0.1840	0.1840	0.1385	0.0822	0.0822	0.1645	0.1645		
Unused land	0.1831	0.1831	0.1263	0.1059	0.1059	0.0603	0.0475	0.0938	0.0938

**Table 3:** Transition area of land-use types from 2000 to 2015.

	Area of Land-Use Types in 2015 (Million ha)								Initial Total Area	Gross Loss
		PF	DL	WL	GL	WB	BL	UL		
**Area of land-use types in 2000 (million ha)**	**PF**	*2.71*	*1.20*	*0.11*	*0.07*	*0.08*	*0.20*	*0.13*	*4.51*	*1.80*
		**4.11**	**0.15**	**0.06**	**0.01**	**0.05**	**0.13**	**0.01**	**4.51**	**0.40**
	**DL**	*1.95*	*23.74*	*2.63*	*1.84*	*0.40*	*1.24*	*0.84*	*32.68*	*8.94*
		**2.95**	**27.22**	**0.81**	**0.41**	**0.17**	**0.88**	**0.25**	**32.69**	**5.47**
	**WL**	*0.19*	*2.78*	*42.61*	*2.68*	*0.21*	*0.17*	*1.48*	*50.11*	*7.50*
		**0.44**	**6.91**	**39.46**	**2.84**	**0.09**	**0.10**	**0.25**	**50.11**	**9.65**
	**GL**	*0.16*	*2.29*	*4.67*	*12.91*	*0.17*	*0.17*	*3.55*	23.91	11.02
		**0.23**	**3.92**	**1.17**	**18.14**	**0.05**	**0.08**	**0.34**	**23.94**	**5.78**
	**WB**	*0.10*	*0.28*	*0.13*	*0.20*	*1.68*	*0.05*	*0.53*	*2.96*	*1.28*
		**0.06**	**0.22**	**0.07**	**0.16**	**2.27**	**0.02**	**0.16**	**2.96**	**0.70**
	**BL**	*0.15*	*0.92*	*0.13*	*0.11*	*0.03*	*1.24*	*0.05*	*2.63*	*1.39*
		**0.22**	**0.89**	**0.06**	**0.09**	**0.02**	**1.33**	**0.03**	**2.63**	**2.31**
	**UL**	*0.29*	*0.81*	*0.49*	*1.19*	*0.26*	*0.07*	*3.90*	*7.00*	*3.10*
		**0.39**	**0.97**	**0.20**	**0.57**	**0.05**	**0.04**	**4.79**	**7.01**	**2.21**
**Final total area**		*5.57*	*32.02*	*50.78*	*19.00*	*2.84*	*3.14*	*10.48*	*123.83*	*35.03*
		**8.40**	**40.28**	**41.82**	**22.23**	**2.70**	**2.59**	**5.82**	**123.83**	**26.52**
**Gross gain**		*2.86*	*8.28*	*8.16*	*6.09*	*1.16*	*1.90*	*6.58*	*35.03*	
		**4.29**	**13.06**	**2.36**	**4.08**	**0.43**	**1.26**	**1.03**	**26.52**	

Note: PF is Paddy field; DL is Dryland; WL is Woodland; GL is Grassland; WB is Water body; BL is Built-up land; UL is Unused land. Values in italics represent the referenced changed area, and values in bold represent the simulated changed area.

**Table 4:** Area and proportion changes of land-use types from 2015 to 2050.

Land Use Types	2015		2050		Area Changes (Million ha)	Proportion Changes (%)
	Area (Million ha)	Proportion (%)	Area (Million ha)	Proportion (%)		
PF	5.57	4.50	7.45	6.01	1.88	1.52
DL	32.02	25.86	31.70	25.60	−0.32	−0.26
WL	50.78	41.01	45.64	36.86	−5.14	−4.15
GL	19.00	15.34	17.26	13.94	−1.74	−1.41
WB	2.84	2.29	3.23	2.61	0.39	0.32
BL	3.14	2.54	4.46	3.60	1.32	1.07
UL	10.48	8.46	14.09	11.38	3.61	2.92

Note: PF is Paddy field; DL is Dryland; WL is Woodland; GL is Grassland; WB is Water body; BL is Built-up land; UL is Unused land.

**Table 5:** Kappa indexes between two land-use maps in 2015.

Land Use Types	Paddy Field	Dryland	Woodland	Grassland	Water Body	Built-Up Land	Unused Land	Total
Kappa index	0.61	0.66	0.85	0.64	0.73	0.69	0.57	0.77

## Data Availability

The data presented in this study are available on request from the corresponding author.
